# A Multistep Approach to Deal With Advanced Heart Failure: A Case Report on the Positive Effect of Cardiac Contractility Modulation Therapy on Pulmonary Pressure Measured by CardioMEMS

**DOI:** 10.3389/fcvm.2022.874433

**Published:** 2022-04-04

**Authors:** Valeria Visco, Cristina Esposito, Michele Manzo, Antonio Fiorentino, Gennaro Galasso, Carmine Vecchione, Michele Ciccarelli

**Affiliations:** ^1^Department of Medicine, Surgery and Dentistry, University of Salerno, Fisciano, Italy; ^2^Cardiology Unit, University Hospital “San Giovanni di Dio e Ruggi D'Aragona”, Salerno, Italy; ^3^Impulse Dynamics Germany GmbH, Frankfurt Am Main, Germany; ^4^Vascular Physiopathology Unit, IRCCS Neuromed, Pozzilli, Italy

**Keywords:** heart failure, case report, Optimizer Smart^®^, cardiac contractility modulation, CardioMEMS, telemonitoring, medical devices, levosimendan

## Abstract

During the last years, the management of heart failure (HF) made substantial progress, focusing on device-based therapies to meet the demands of this complex syndrome. In this case report, we present a multistep approach to deal with HF. Specifically, we report the first patient subjected to the implantation of both Optimizer Smart^®^ (Impulse Dynamics Inc., Marlton, NJ, USA) and CardioMEMS devices. A 72-year-old male patient with HF and reduced ejection fraction (HFrEF) was admitted to our cardiology department in January 2021, following a progressive shortening of the time between hospitalizations for levosimendan infusions. Specifically, the patient was monitored daily by CardioMEMS, and a strategy of levosimendan infusions guided by the device had been adopted. He was also a carrier of MitraClips and cardiac resynchronization therapy defibrillator (CRT-D) and had optimized HF medical therapy. In January 2021, the patient implanted Optimizer Smart^®^ device for cardiac contractility modulation (CCM) therapy because of poor response to therapy and elevated pulmonary artery pressure (PAP). CCM significantly reduced PAP values following discharge (systolic PAP 33.67 ± 2.92 vs. 40.6 ± 3.37 mmHg, diastolic PAP 14.5 ± 2.01 vs. 22.5 ± 2.53 mmHg, mean PAP 22.87 ± 2.20 vs. 30.9 ± 2.99 mmHg, HR 60.93 ± 1.53 vs. 80.83 ± 3.66 bpm; *p* < 0.0001), with persisting effect at 9 months. The usefulness of CCM is objectively demonstrated for the first time by continuous invasive monitoring of PAP by CardioMEMS, which can suggest the correct timing for CCM implantation.

## Introduction

Heart failure (HF) shows phases of exacerbation interrupted by periods of clinical stability. Despite many pharmacological advances, morbidity and mortality in HF remain an important burden to patients, caregivers, and national healthcare systems (1–3). Advanced HF, defined as severe symptoms despite optimal medical therapy (OMT) and device, affects up to 25% of patients with HF (4). Treatments for such patients are limited. Consequently, during the last years, the management of HF made substantial progress, focusing on device-based therapies to meet the demands of this complex syndrome. Specifically, in the management of this syndrome, therapeutic strategies usually aim for improved outcomes in terms of reduced mortality and fewer unplanned HF hospitalizations (5).

**Figure 1:**
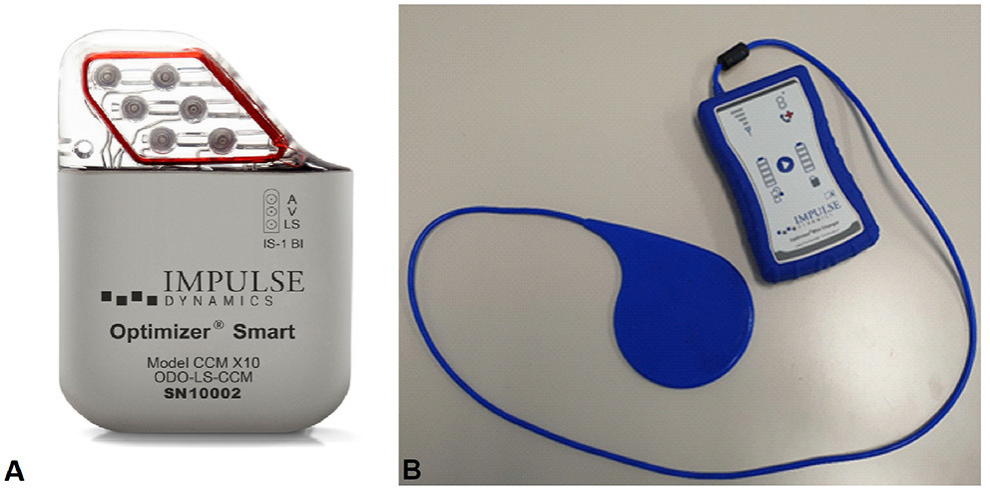
Optimizer Smart^®^ system. **(A)** The Optimizer Smart^®^ Implantable Pulse Generator (IPG) unit. **(B)** Optimizer Smart Charger System. Patients can recharge the IPG unit using a portable, home-based charger system, without supervision. Charging the device on a weekly basis assures it has enough energy to provide the prescribed dose of CCM therapy.

On the one hand, the CardioMEMS (Abbott Medical, Inc., Abbott Park, Illinois, USA) is an implantable device positioned in the pulmonary artery and is able to detect higher cardiac filling pressures, an objective measure of “hemodynamic congestion,” estimated to rise more than 2 weeks before the onset of symptomatic congestion (6, 7); this device is applicable in patients with chronic HF in functional New York Heart Association (NYHA) Class III with at least one admission for HF in the past year (class of recommendation IIb according to HF ESC Guideline 2021) (2). On the other hand, cardiac contractility modulation (CCM) therapy has emerged as a hopeful device treatment in patients with HF not indicated for cardiac resynchronization therapy (5). Specifically, CCM is an electrical therapy that consists of biphasic pulses of relatively high voltage being delivered to the right ventricular (RV) septum *via* a small implantable pulse generator in the absolute refractory period of the myocardium (8–10) (Figure 1). In this report, we present a multistep approach to deal with HF.

## Case Presentation

A 72-year-old Caucasian male patient with HF and reduced ejection fraction (HFrEF) was admitted to our cardiology department in January 2021, following a progressive shortening of the time between hospitalizations for levosimendan infusions. Specifically, the patient had no symptoms or signs of HF exacerbation; however, he had a reduction in exercise tolerance (symptoms graded as Class III of the NYHA Functional Classification). Physical examination showed vesicular breath sounds, no peripheral edema, normal peripheral pulses, and systolic murmur in the mitral area. Moreover, blood tests showed brain natriuretic peptide (BNP) 1,093 pg/ml, hemoglobin 10 g/dl, and estimated glomerular filtration rate (eGFR) 25 ml/min/1.73 m^2^. The patient's comorbidities comprised chronic kidney disease stage 4 with secondary anemia, dyslipidemia, carotid atheromasia, paroxysmal atrial fibrillation, hypothyroidism, chronic obstructive pulmonary disease, sizeable inguinal hernia, arterial hypertension, and obesity.

## Past Medical History

His medical history started in 2004, when he was diagnosed with primary hypokinetic dilated cardiomyopathy (EF 42%) in the absence of significant coronary artery disease. Despite the OMT, from 2004 to 2015, he had more than 20 hospitalizations for HF exacerbation with a gradual reduction of EF. In 2015, he underwent biventricular implantable cardioverter defibrillator (CRT-D) implantation for the evidence of EF <35%, while in 2017, he was treated by positioning of MitraClips (Abbott Laboratories, Menlo Park, California, USA) for severe mitral regurgitation. In the same year, the patient was enrolled in our HF clinic and further optimized HF therapy with the addition of Sacubitril/Valsartan 24/26 mg b.i.d. However, despite the OMT, from October 2017 to 2019, the patient underwent numerous hospitalizations. Then, in June 2019, a strategy of levosimendan infusions guided by CardioMEMS was implemented to obtain hemodynamic stability in the patient for a more extended period. Consequently, the patient was monitored daily by the device, and when home therapeutic changes were not sufficient, the patient was contacted for hospitalization and levosimendan infusion. From June 2019 to January 2021, the patient had only 5 hospitalizations scheduled for levosimendan infusion and only one for HF exacerbation. In January 2021, following a progressive shortening of the time between hospitalizations for levosimendan infusions, the patient was hospitalized in our Cardiology Unit to change the management strategy.

## Investigations

An echocardiogram (January 2021) showed a dilated left ventricle (LV end-diastolic volume 197 ml) with impaired LV systolic function (EF 25%) and global hypokinesia, LV diastolic dysfunction with elevation in LV filling pressure, moderate mitral regurgitation and presence of MitraClips, dilated left atrium (volume 54 ml/m^2^), a dilated right ventricle (RVD 57 mm) with preserved function (TAPSE 18 mm), increased pulmonary artery systolic pressure (41 mmHg), dilated inferior vena cava (IVC), and no collapse. Normal sinus rhythm was observed on the electrocardiogram.

## Management

### Procedure

The patient received the typical 24-h intravenous infusion of levosimendan (0.1 μg/kg/min); subsequently, to reduce HF-related hospitalizations and to improve quality of life, we decided to implant a CCM therapy device. After signing informed consent, the Optimizer Smart (Impulse Dynamics Inc., Marlton, NJ, USA) for the delivery of CCM therapy was implanted with fluoroscopic guidance.

**Figure 2:**
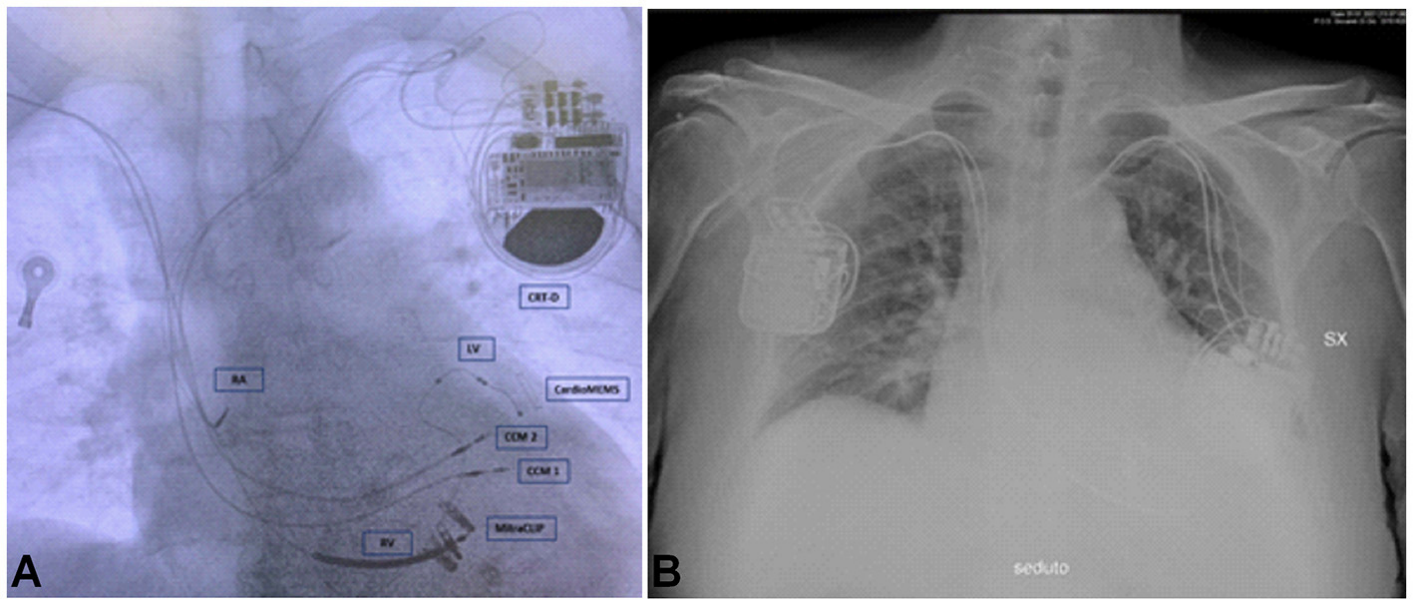
Chest X-ray of the patient. **(A)** The figure shows two CCM leads, three CRT-D leads, CardioMEMS, and MitraClips. **(B)** The figure shows CCM device on the right and CRT-D on the left. CRT-D, cardiac resynchronization therapy – defibrillator device; CCM 1, lead 1 CCM; CCM 2, lead 2 CCM; RV, right ventricular lead for CRT-D; LV, left ventricular lead for CRT-D; RA, right atrial lead for CRT-D.

**Figure 3:**
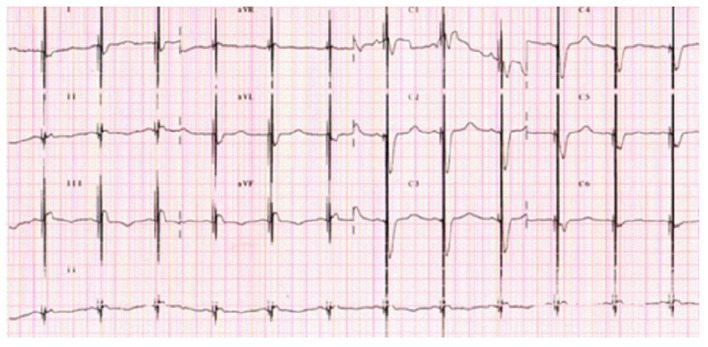
A 12-lead ECG with visible spikes of high-energy impulses from CCM therapy.

Because a CRT-D was already present in the left prepectoral area, Optimizer Smart implant was performed *via* a contralateral right-sided access. Current devices require the implantation of two standard pacing leads in the RV. Therefore, a device pocket was fashioned in the prepectoral area, and the leads (Tendril 2088TC – 58, Abbott) were sequentially implanted using two peel-away sheaths introduced over the guide wires. For each lead, mapping to optimize location was performed; particularly, the RV lead tips were placed along the septal wall at 2 cm apart (8). Finally, the septal position of the lead was confirmed by oblique fluoroscopy views and pacing ECG patterns. A testing for device–device interactions was performed to ascertain oversensing of the previously implanted CRT-D, given the large voltage signal (7.5 V/22-ms duration) produced by CCM (8). The Optimizer unit was then attached to the leads and located in the pocket (Figure 2). The Optimizer pulse generator needs manual transcutaneous recharging by the patient: routine recharging is done weekly and needs about 1 h to complete (11). In our case, the CCM therapy was scheduled for 10 h/day (Figure 3).

### Follow-Up

**Figure 4:**
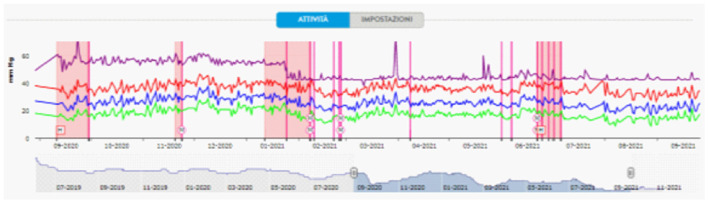
Pulmonary artery pressure tracing detected by CardioMEMS. Starting from September 2020, there was a progressive shortening of the time between hospitalizations (pink squares) for levosimendan infusions. Pulmonary artery pressure tracing showing a mean diastolic PA drop after Optimizer Smart^®^ implantation (January 2021). M and/or purple lines, remote therapy modifications; H, hospitalization.

The PA pressure values on monitoring in the 30 days following discharge were significantly lower than in the previous hospitalization (PAPs 33.67 ± 2.92 vs. 40.6 ± 3.37 mmHg, PAPd 14.5 ± 2.01 vs. 22.5 ± 2.53 mmHg, PAPm 22.87 ± 2.20 vs. 30.9 ± 2.99 mmHg, HR 60.93 ± 1.53 vs. 80.83 ± 3.66 bpm; *p* < 0.0001), and this effect persisted even after 9 months (Figure 4).

Specifically, after January 2021, the cardiologist continued to monitor the patient, making therapy adjustments according to PAPd, and no further levosimendan infusions or hospitalizations were made between January 2021 and June 2021. The patient was evaluated 4 weeks after implantation; at that visit, the pulse generator was interrogated to assess the percentage of beats receiving CCM impulse delivery to ensure the adequacy of CCM parameter programming (Supplementary Figure 1). Moreover, a 5-level EQ-5D version (EQ-5D-5L) questionnaire about quality of life was performed: the score increased from 79 (before) to 91 (4 weeks after implantation of Optimizer Smart^®^).

## Discussion

In this study, we report the case of a patient with advanced HF, whose quality of life was strictly invalidated by the multiple HF exacerbations. Our case supports a step-by-step approach to treat heart problems, with the help of innovative devices.

The CCM has been studied in preclinical and clinical studies over the past years and has now been found to benefit a population of patients characterized as having: NYHA Class III–IV despite the OMT, EF 25–45%, and electrocardiogram QRS complex duration <130 ms (2, 12, 13). Moreover, some publications have verified the safety and effectiveness of the CCM in CRT nonresponder patients: the implants are justified in those patients where symptoms persist despite OMT (14, 15).

Specifically, in this report, we present the first patient experience with levosimendan infusion led by CardioMEMS and the subsequent Optimizer Smart^®^ device implantation; consequently, the usefulness of CCM is objectively demonstrated for the first time by continuous invasive monitoring of pulmonary arterial pressures.

The CCM is a novel HF device therapy that acts through a high-energy, nonexcitatory biphasic signal in the absolute ventricular refractory period of the heart cycle supplied by two leads placed on the RV septum (5). Contrary to other electrical stimulation, such as pacemaker therapy (CRT-P) or implantable cardioverter defibrillators (CRT-D), CCM does not affect the cardiac rhythm directly.

In particular, CCM improves calcium handling, reverses the fetal myocyte gene program associated with HF, and fosters reverse remodeling (5, 9, 16), through molecular remodeling and restoration of intracellular Ca2+ regulatory proteins, such as SERCA2a, phospholamban, and RyR2 (17). Furthermore, the HF gene expression profile was found to be reversed (toward normality) in patients who received the device (17). Moreover, the CCM therapy does not increase myocardial oxygen consumption but instead improves functional capacity (as measured by peak VO2 and 6MWT distance) and quality of life (as measured by MLWHFQ score) in patients with HF (5). In addition, the beneficial effects of CCM appear to be independent of HF etiology and QRS duration (18–21). These positive effects of CCM retain up to 3 years of follow-up, with a reduction in HF hospitalizations and improvements in functional status and quality of life extended at least through 24 months (22).

Finally, the FIX-HF-5C study showed an 89.7% complication-free rate, which achieved the primary safety endpoint (20). Specifically, the safety/adverse events included 5 events of lead dislodgements, 1 deep vein thrombosis, and 1 generator erosion resulting in pocket stimulation that required pocket revision and replacement of pacemaker leads (20). Subsequently, in the FIX-HF-5C2 study, there were decreased Optimizer-related adverse events with the 2-lead system compared with the previous 3-lead system (0% vs. 8%; *p* = 0.03) (23); specifically, complication rates mirror those of dual-chamber pacemaker procedures (13).

## Conclusion

In our case report, following the Optimizer Smart^®^ implantation, we observed a reduction of the HF hospitalizations (scheduled and not scheduled), reduction of the diuretic dosage, and reduction in pulmonary artery pressures. The effective decrease of pulmonary pressures brought by the Optimizer Smart^®^ implantation was recorded and witnessed by the CardioMEMS device.

Consequently, our case supports the “real-world” effectiveness and feasibility of this multistep approach for the management of advanced HF.

## Data Availability Statement

The raw data supporting the conclusions of this article will be made available by the authors, without undue reservation.

## Ethics Statement

Ethical review and approval was not required for the study on human participants in accordance with the local legislation and institutional requirements. The patients/participants provided their written informed consent to participate in this study. Written informed consent was obtained from the individual(s) for the publication of any potentially identifiable images or data included in this article.

## Author Contributions

VV and MC: conceptualization, formal analysis, investigation, resources, writing–original draft, and writing–review and editing. VV, CE, MM, GG, CV, and MC: data curation. AF: writing–review and editing. All authors have discussed, read, and approved the submission of this manuscript for publication.

## Conflict of Interest

AF is a full-time employee of Impulse Dynamics where Optimizer Smart^®^ was discovered and developed. The remaining authors declare that the research was conducted in the absence of any commercial or financial relationships that could be construed as a potential conflict of interest.

## Publisher's Note

All claims expressed in this article are solely those of the authors and do not necessarily represent those of their affiliated organizations, or those of the publisher, the editors and the reviewers. Any product that may be evaluated in this article, or claim that may be made by its manufacturer, is not guaranteed or endorsed by the publisher.

## Supplementary Material

Supplementary Figure 1 Optimizer Smart^®^ interrogation after 4 weeks. The percentage of beats receiving CCM impulse delivery was 99.9%.

Supplementary Table 1 Timeline of the case. HF, heart failure; EF, ejection fraction, CRT-D, biventricular ICD; OMT, optimal medical therapy; PAP, pulmonary artery pressure.
