# Supplementary figures and images for: A Multistep Approach to Deal With Advanced Heart Failure: A Case Report on the Positive Effect of Cardiac Contractility Modulation Therapy on Pulmonary Pressure Measured by CardioMEMS
Source: Front Cardiovasc Med. 2022 Apr 4;9:874433. doi: 10.3389/fcvm.2022.874433 (PMC9013826; doi:10.3389/fcvm.2022.874433)

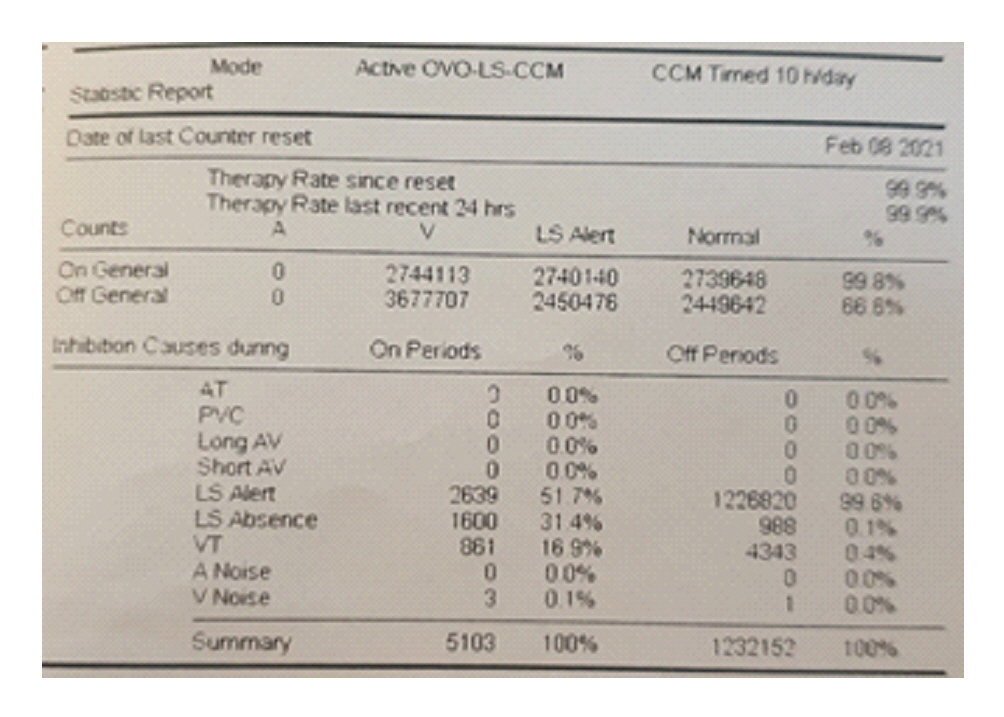

Supplement: Supplementary Figure 1 — Optimizer Smart® interrogation after 4 weeks. The percentage of beats receiving CCM impulse delivery was 99.9%. [file Image_1.JPEG]
