# Supplementary material for: A Multistep Approach to Deal With Advanced Heart Failure: A Case Report on the Positive Effect of Cardiac Contractility Modulation Therapy on Pulmonary Pressure Measured by CardioMEMS
Source: Front Cardiovasc Med. 2022 Apr 4;9:874433. doi: 10.3389/fcvm.2022.874433 (PMC9013826; doi:10.3389/fcvm.2022.874433)
Supplement: Supplementary Table 1 — Timeline of the case. HF, heart failure; EF, ejection fraction, CRT-D, biventricular ICD; OMT, optimal medical therapy; PAP, pulmonary artery pressure. [file Table_1.DOCX]

**TIMELINE**

| Date | Events |
| --- | --- |
| 2004 | Diagnosed with primary hypokinetic dilated cardiomyopathy (EF 42%). |
| 2004-2015 | More than 20 hospitalizations for HF exacerbation with a progressive reduction of the ejection fraction (EF). |
| 2015 | **EF < 35%: CRT-D implantation for primary prevention.** |
| September 2017 | **MitraClips placement.** |
| October 2017 | **The patient started HF optimal medical therapy (OMT).** |
| October 2017-May 2019 | The patient went through numerous exacerbations, despite OMT. |
| June 2019 | **CardioMEMS implantation and decision to start levosimendan infusions guided by CardioMEMS** |
| September 2020 | Progressive shortening of the time between hospitalizations for levosimendan infusions. |
| January 2021 | **Optmizer Smart implantation: PAP tracing showing a mean diastolic PAP drop.** |
| September 2021 | PAP drop persists even after 9 months. |
