# Supplementary material for: A Multistep Approach to Deal With Advanced Heart Failure: A Case Report on the Positive Effect of Cardiac Contractility Modulation Therapy on Pulmonary Pressure Measured by CardioMEMS
Source: Front Cardiovasc Med. 2022 Apr 4;9:874433. doi: 10.3389/fcvm.2022.874433 (PMC9013826; doi:10.3389/fcvm.2022.874433)
Supplement: Supplementary file 3 [file Data_Sheet_1.PDF]

CARE case report guidelines

CARE Checklist of information to include when writing a case report

(cc) BY-NC-ND

Topic	Item	Checklist item description	Reported on Line
Title	1	The diagnosis or intervention of primary focus followed by the words "case report"	0, TITLE
Key Words	2	2 to 5 key words that identify diagnoses or interventions in this case report, including "case report"	16-17
Abstract (no references)	3a	Introduction: What is unique about this case and what does it add to the scientific literature?	20-24
	3b	Main symptoms and/or important clinical findings	25-29
	3c	The main diagnoses, therapeutic interventions, and outcomes	29-34
	3d	Conclusion—What is the main "take-away" lesson(s) from this case?	35-36
Introduction	4	One or two paragraphs summarizing why this case is unique (**may include references**)	38-57
Patient Information	5a	De-identified patient specific information.	59-60
	5b	Primary concerns and symptoms of the patient.	61-63
	5c	Medical, family, and psycho-social history including relevant genetic information	71-86
	5d	Relevant past interventions with outcomes	71-86
Clinical Findings	6	Describe significant physical examination (PE) and important clinical findings.	63-64
Timeline	7	Historical and current information from this episode of care organized as a timeline	tAB 1 SUPP
Diagnostic Assessment	8a	Diagnostic testing (such as PE, laboratory testing, imaging, surveys).	64-66, 88-93
	8b	Diagnostic challenges (such as access to testing, financial, or cultural)	n/a
	8c	Diagnosis (including other diagnoses considered)	n/a
	8d	Prognosis (such as staging in oncology) where applicable	n/a
Therapeutic Intervention	9a	Types of therapeutic intervention (such as pharmacologic, surgical, preventive, self-care)	96-116
	9b	Administration of therapeutic intervention (such as dosage, strength, duration)	118-126
	9c	Changes in therapeutic intervention (with rationale)	60-61
Follow-up and Outcomes	10a	Clinician and patient-assessed outcomes (if available)	118-126
	10b	Important follow-up diagnostic and other test results	118-126
	10c	Intervention adherence and tolerability (How was this assessed?)	N/A
	10d	Adverse and unanticipated events	N/A
Discussion	11a	A scientific discussion of the strengths AND limitations associated with this case report	128-150
	11b	Discussion of the relevant medical literature **with references**.	128-150
	11c	The scientific rationale for any conclusions (including assessment of possible causes)	128-150
	11d	The primary "take-away" lessons of this case report (without references) in a one paragraph conclusion	152-157
Patient Perspective	12	The patient should share their perspective in one to two paragraphs on the treatment(s) they received	n/a
Informed Consent	13	Did the patient give informed consent? Please provide if requested	Yes [x] No [ ]
